# Control of cytokine mRNA degradation by the histone deacetylase inhibitor ITF2357 in rheumatoid arthritis fibroblast-like synoviocytes: beyond transcriptional regulation

**DOI:** 10.1186/s13075-018-1638-4

**Published:** 2018-07-20

**Authors:** Chiara Angiolilli, Pawel A. Kabala, Aleksander M. Grabiec, Marzia Rossato, Wi S. Lai, Gianluca Fossati, Paolo Mascagni, Christian Steinkühler, Perry J. Blackshear, Kris A. Reedquist, Dominique L. Baeten, Timothy R. D. J. Radstake

**Affiliations:** 1 Laboratory of Translational Immunology and Department of Rheumatology and Clinical Immunology, University Medical Center Utrecht, Utrecht, The Netherlands; 2 Amsterdam Rheumatology and Immunology Center, Department of Clinical Immunology and Rheumatology and Department of Experimental Immunology, Academic Medical Center/University of Amsterdam, Amsterdam, The Netherlands; 3 Department of Microbiology, Faculty of Biochemistry, Biophysics and Biotechnology, Jagiellonian University, Kraków, Poland; 4 Functional Genomics Center, University of Verona, Verona, Italy; 5 Signal Transduction Laboratory, National Institute of Environmental Health Sciences, Research Triangle Park, NC 27709 USA; 6 Italfarmaco Research and Development, Cinisello Balsamo, Italy

**Keywords:** Rheumatoid arthritis, Fibroblast-like synoviocytes, Inflammation, mRNA stability, Tristetraprolin, Histone deacetylase inhibitor, ITF2357

## Abstract

**Background:**

Histone deacetylase inhibitors (HDACi) suppress cytokine production in immune and stromal cells of patients with rheumatoid arthritis (RA). Here, we investigated the effects of the HDACi givinostat (ITF2357) on the transcriptional and post-transcriptional regulation of inflammatory markers in RA fibroblast-like synoviocytes (FLS).

**Methods:**

The effects of ITF2357 on the expression and messenger RNA (mRNA) stability of IL-1β-inducible genes in FLS were analyzed using array-based qPCR and Luminex. The expression of primary and mature cytokine transcripts, the mRNA levels of tristetraprolin (TTP, or ZFP36) and other AU-rich element binding proteins (ARE-BP) and the cytokine profile of fibroblasts derived from *ZFP36*^*+/+*^ and *ZFP36*^*−/−*^ mice was measured by qPCR. ARE-BP silencing was performed by small interfering RNA (siRNA)-mediated knockdown, and TTP post-translational modifications were analyzed by immunoblotting.

**Results:**

ITF2357 reduced the expression of 85% of the analyzed IL-1β-inducible transcripts, including cytokines (*IL6*, *IL8*), chemokines (*CXCL2*, *CXCL5*, *CXCL6*, *CXCL10*), matrix-degrading enzymes (*MMP1*, *ADAMTS1*) and other inflammatory mediators. Analyses of mRNA stability demonstrated that ITF2357 accelerates *IL6*, *IL8*, *PTGS2* and *CXCL2* mRNA degradation, a phenomenon associated with the enhanced transcription of TTP, but not other ARE-BP, and the altered post-translational status of TTP protein. TTP knockdown potentiated cytokine production in RA FLS and murine fibroblasts, which in the latter case was insensitive to inhibition by ITF2357 treatment.

**Conclusions:**

Our study identifies that regulation of cytokine mRNA stability is a predominant mechanism underlying ITF2357 anti-inflammatory properties, occurring via regulation of TTP. These results highlight the therapeutic potential of ITF2357 in the treatment of RA.

**Electronic supplementary material:**

The online version of this article (10.1186/s13075-018-1638-4) contains supplementary material, which is available to authorized users.

## Background

Rheumatoid arthritis (RA) is a chronic immune-mediated inflammatory disease, characterized by the excessive activation of the immune system and the uncontrolled production of cytokines and other inflammatory mediators in synovial joints. Cytokines such as tumor necrosis factor (TNF) and interleukin (IL)-1β produced by macrophages and lymphocytes infiltrating the synovial tissue lead to the abnormal activation of fibroblast-like synoviocytes (FLS), which in turn causes bone and cartilage deterioration [1]. Regulation of inflammatory cytokines occurs at multiple levels and results from the intricate modulation of epigenetic regulatory mechanisms, activation of intracellular signaling pathways, control of messenger RNA (mRNA) stability and protein translation. The correct regulation of mRNA decay is critical for immune homeostasis, as it allows cells to quickly adjust the expression of inflammatory mediators, the overproduction of which could adversely affect the organism [2]. Conditions that interfere with stability of mRNA are associated with diverse diseases, including chronic inflammation and cancer [3].

Adenosine uridine (AU)-rich elements (AREs) represent one of the largest and most important groups of *cis*-acting mRNA stability determinants. AREs allow the recruitment of *trans*-acting ARE binding proteins (ARE-BP), which in turn mediate mRNA degradation [4]. Several human ARE-BP have been identified, such as tristetraprolin (TTP, or ZFP36), TTP family members BRF1 (ZFP36L1) and BRF2 (ZFP36L2), AU-rich binding factor-1 (AUF1, or HNRNPD), KH-type splicing regulatory protein (KHSRP), and Hu antigen R (HuR, or ELAVL1). The majority of ARE-BP promote the recruitment of ARE-containing mRNAs to the exosomes for eventual degradation, although some, such as HuR and Hu family members, act as mRNA stabilizing factors [5].

The expression of several ARE-BP was found to be dysregulated in RA, and their silencing shown to affect key regulatory mechanisms in arthritis pathogenesis, both in vitro and in vivo [6–10]. To date, TTP is the ARE-BP that has been best characterized and associated with RA development and disease progression [11, 12]. TTP expression is altered in patients with synovium affected by RA [11] and TTP-deficient mice display a severe inflammatory phenotype that includes synovial pannus formation and erosive arthritis [10]. Remarkably, overexpression of endogenous TTP or mutations at TTP phosphorylation sites protect mice in experimental models of arthritis [11, 13].

Growing interest in the modulation of ARE-BP in RA pathology has thus promoted the search for novel inhibitory compounds that can reverse the aberrant expression and function of ARE-BP. Inhibitors of the mitogen-activated protein kinase (MAPK) p38, a critical regulator of the phosphorylation status and activity of multiple ARE-BP, have been extensively used to dampen uncontrolled production of pro-inflammatory cytokines resulting from dysregulated mRNA decay [14]. However, p38 inhibitors are currently not approved for RA treatment due to molecule-related adverse events, such as cutaneous toxicity, and limited clinical efficacy [15, 16]. HDACi represent a novel class of small molecule drugs that have shown promising results in vitro and in vivo in models of RA and immune-related diseases [17, 18] and have demonstrated initial clinical efficacy in the treatment of systemic-onset juvenile idiopathic arthritis [19]. Although the primary mechanism of action of HDACi is proposed to rely on the regulation of chromatin opening and transcription, studies have reported that HDACi can impair cytokine mRNA expression despite favoring their transcriptional activation [20, 21]. We previously reported that pan-specific HDACi ITF2357 (Givinostat) and trichostatin A (TSA) prevented IL-6 production in RA FLS and macrophages by promoting accelerated degradation of *IL6* mRNA [22].

In this study, we aimed to dissect the transcriptional and post-transcriptional regulation of cytokine mRNA expression by ITF2357, and to identify whether, and through which mechanisms, HDACi can restore the balance in mRNA-stability mechanisms that are deregulated in RA.

## Methods

### Patient material and FLS isolation

FLS were derived from synovial tissue specimens obtained from patients with RA by needle arthroscopy, as previously described [23], cultured in medium supplemented with 10% fetal bovine serum (FBS, Invitrogen), and used between passages 4 and 10. All patients fulfilled the criteria for the classification of RA and had active disease including clinical arthritis of the joint from which the synovial biopsies were obtained [24].

### FLS treatment and stimulation

FLS were cultured overnight in Dulbecco’s Modified Eagle Medium (DMEM, Life Technologies) containing 1% FBS prior to incubation with cytokines. Cells were pre-incubated for 30 min with either 250 nM pan-HDAC inhibitor ITF2357 (Italfarmaco) or 5 μM p38 inhibitor (SB202190, Sigma) and stimulated with 1 ng/ml IL-1β (R&D Systems). Information about the specificity of the HDACi is published [25].

### RNA extraction and gene expression profiling

RNeasy Micro Kit (Qiagen) was used for RNA extraction. Quantity and purity of RNA was assessed using a Nanodrop spectrophotometer (Nanodrop Technologies). RNA was reverse-transcribed using a First-Strand complementary DNA (cDNA) synthesis kit (Thermo Scientific) and quantitative (q)PCR was performed using Sybr Select PCR Master Mix (Applied Biosystems). For qPCR array analysis, RNA was reverse-transcribed using an RT^2^ HT First Strand Kit (Qiagen), cDNA was mixed with Sybr Green qPCR Master Mix (Qiagen) and expression of 83 genes involved in FLS activation was analyzed using RT^2^ Profiler customized qPCRarrays. qPCR reactions were performed on a StepOnePlus Real-Time PCR System (Applied Biosystems) and relative mRNA expression was calculated using StepOne Software V.2.1 (Applied Biosystems). Sequences of the primers used are listed in Additional file 4. The ratio between the gene of interest and the expression of human *GAPDH* or murine *ACTB* housekeeping genes, or the expression of five housekeeping genes (*B2M*, *HPRT1*, *RPL13A*, *GAPDH* and *ACTB*) was calculated for qPCR and qPCR arrays, respectively.

### Protein extraction and immunoblotting

FLS were lysed in Laemmli’s buffer and protein content was quantified with a BCA Protein Assay Kit (Pierce). Equivalent amounts of total protein lysate were then mixed with loading buffer and boiled at 95 °C for 5 min. Proteins were resolved by electrophoresis on either 4–12% Bis-Tris SDS NuPAGE gels (Invitrogen) for 1 h at constant 200 V, or on 10% SDS-PAGE gels for 5 h at constant 70 V for better separation of immunoreactive bands ranging between 26 and 55 kDa. Gels were transferred to polyvinylidene difluoride (PVDF) membranes (Bio-Rad Laboratories), membranes were blocked in Tris-buffered saline (pH 8.0) containing 0.05% Tween-20 (Bio-Rad) and 4% milk (Bio-Rad), washed and probed overnight at 4 °C with antibodies recognizing TTP (Cell Signaling), histone 3 (H3) (Cell Signaling) or tubulin (Sigma-Aldrich). After washing, membranes were incubated with horseradish peroxidase (HRP)-conjugated swine anti-rabbit or goat anti-mouse immunoglobulin secondary antibody (Dako), and protein visualization was performed using a ChemiDoc MP system (Bio-Rad).

### Luminex assay

RA FLS were left unstimulated or were treated with 250 nM ITF2357 for 30 min prior to stimulation with 1 ng/ml IL-1β for 24 h. Supernatants were harvested and IL-8, matrix metalloproteinase (MMP)-3, CXCL-10, CXCL-5 and CXCL-6 protein secretion determined by Luminex (BioRad) according to the manufacturer’s instructions at the core facility of the Academic Medical Center (AMC).

### Analysis of mRNA stability

FLS were left unstimulated or were treated with 250 nM ITF2357 for 30 min prior to stimulation with 1 ng/ml IL-1β. After 2 h of stimulation culture medium was discarded, cells were washed and fresh medium containing 10 μg/ml actinomycin D (ActD) (Sigma-Aldrich) was added. Cells were then harvested at 0, 2 and 5 h following the addition of ActD, RNA was isolated, and the rates of mRNA degradation in the presence or absence of HDACi assessed using a customized RT^2^ Profiler™ PCR Array set (SABiosciences) as described above. Transcripts displaying at least 1.5-fold change in the rate of degradation, compared to IL-1β-stimulated controls were analyzed.

### Lambda phosphatase treatment

FLS were lysed in 1 × NEBuffer (New England Biolabs) supplemented with 1 mM MnCl_2_. Cell lysate was incubated on ice for 20 min, spun down, and supernatant was collected and incubated with 10,000 U/ml lambda (λ) phosphatase (New England Biolabs) at 30 °C for 30 min. Protein lysate was added to loading buffer and boiled at 95 °C for 5 min, and further processed for immunoblotting as described above.

### siRNA transfection

RA FLS were transfected using DharmaFECT1 (Thermo Scientific). The day before transfection, cells were incubated with DMEM containing 10% FBS which was then replaced with OPTI-MEM serum-reduced medium. AUF1, BRF1, BRF2, KHSRP, HuR and TTP specific small interfering RNA (siRNA) (20 nM) and control non-targeting siRNA (20 nM), (Thermo Scientific) were mixed with DharmaFECT1 and incubated for 20 min at room temperature prior to transfection; 24 h after transfection, medium was replaced with DMEM containing 10% FBS and this was left for another 24 h.

### TTP wild-type and knockout MEF

Mouse embryonic fibroblasts (MEF) were derived from littermate E14.5 *Zfp36 (TTP) +/+* and *Zfp36 (TTP)− /−* embryos, as previously described [10]. *Zfp36−/−* mice were generated by inserting a targeting vector containing a neomycin resistance gene (neo) in the TTP protein-coding region, which generated multiple stop codons and precluded synthesis of the functional protein. MEFs were maintained in medium containing 10% FBS, 100 U/ml penicillin, 100 μg/ml streptomycin, and 2mM L-glutamine. *Zfp36−/−* cells were regularly maintained for one passage in feeding medium containing 0.3 mg/ml of the selection antibiotic Geneticin (G418, Thermo Fisher Scientific).

### Statistical analysis

Data are presented as mean ± SEM unless otherwise indicated. One-way analysis of variance (ANOVA) was used for analyzing sets of data requiring multiple comparisons. Wilcoxon matched pairs test and the ratio *t* test was used for all other paired comparisons. Data were analyzed using GraphPad software 7 with *p* values < 0.05 considered statistically significant.

## Results

### ITF2357 rapidly suppresses the expression of IL-1β-induced inflammatory genes

**Fig. 1:**
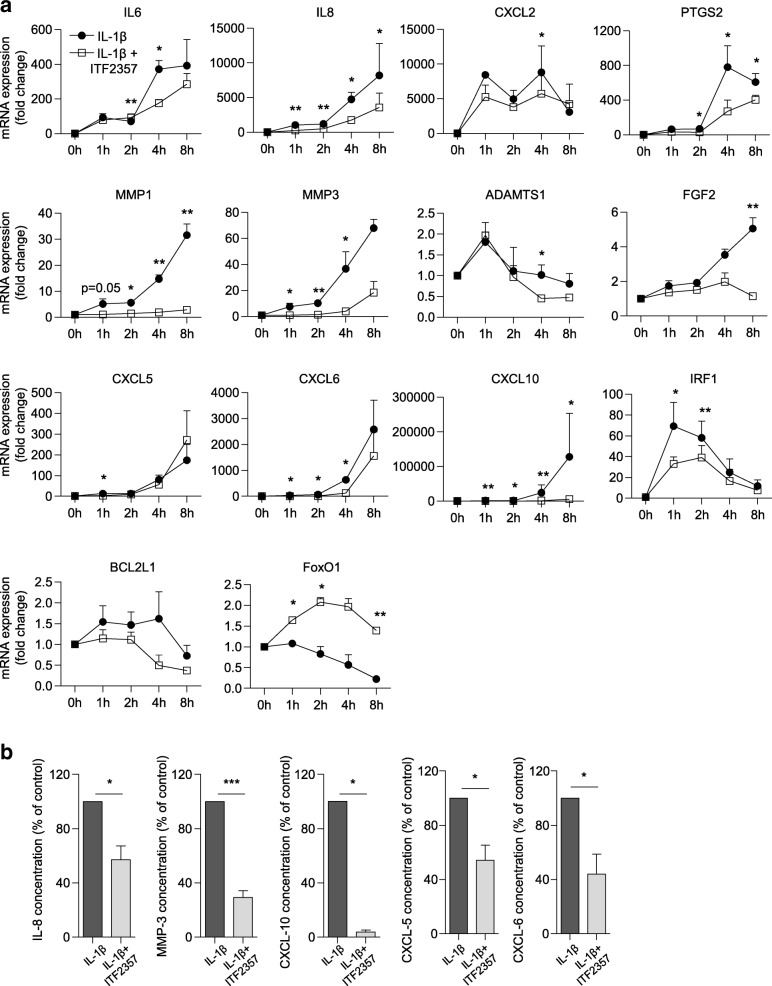
ITF2357 suppresses the expression of IL-1β-responsive genes. **a** Rheumatoid arthritis (RA) fibroblast-like synoviocytes (FLS) (*n* = 3) were either left untreated or were treated with ITF2357 prior to incubation with IL-1β for the indicated time. Temporal changes in mRNA accumulation of IL-1β-inducible genes were monitored using a customized qPCR array system. Data are presented as fold changes in mRNA levels compared to unstimulated cells in the presence or absence of ITF2357 Differences in fold changes between IL-1β and IL-1β + ITF2357 conditions, for each time point, were analyzed by ratio *t* test: *p < 0.05, **p < 0.01. **b** RA FLS were either left untreated or were treated with ITF2357 prior to incubation with IL-1β for 24 h. FLS supernatant was harvested and levels of IL-8, matrix metalloproteinase (MMP)-3, CXCL-10 (*n* = 6) CXCL-5 and CXCL-6 (*n* = 5) were measured by Luminex. Protein concentrations were normalized to 100% in each experiment for samples not treated with histone deacetylase inhibitor and expressed as the percentage of control:*p < 0.05, ***p < 0.001, ratio *t* test

We and others have shown that the pan-HDACi ITF2357 is a potent suppressor of genes regulating inflammatory activation, adhesion, angiogenesis, cell survival and extracellular matrix degradation [25–27]. Specifically, by screening a broad subset of genes relevant to disease pathology in RA, we found that treatment with ITF2357 reduced the expression of the majority of genes responsive to IL-1β stimulation in RA FLS [26]. To gain more insight into temporal changes in gene expression in the presence of the HDACi, we analyzed the kinetics of mRNA regulation of 83 selected genes using customized qPCR arrays (Fig. 1a and Additional file 1). ITF2357 reduced the expression of 85% of the analyzed transcripts, regardless of the kinetics of gene induction after IL-1β stimulation. As earlier shown for *IL6* [22], the reduction observed in cytokine mRNA accumulation after ITF2357 treatment corresponded to changes at the protein level (Fig. 1b).

### Reduced expression of a subset of ITF2357-regulated genes is associated with mRNA decay

**Fig. 2:**
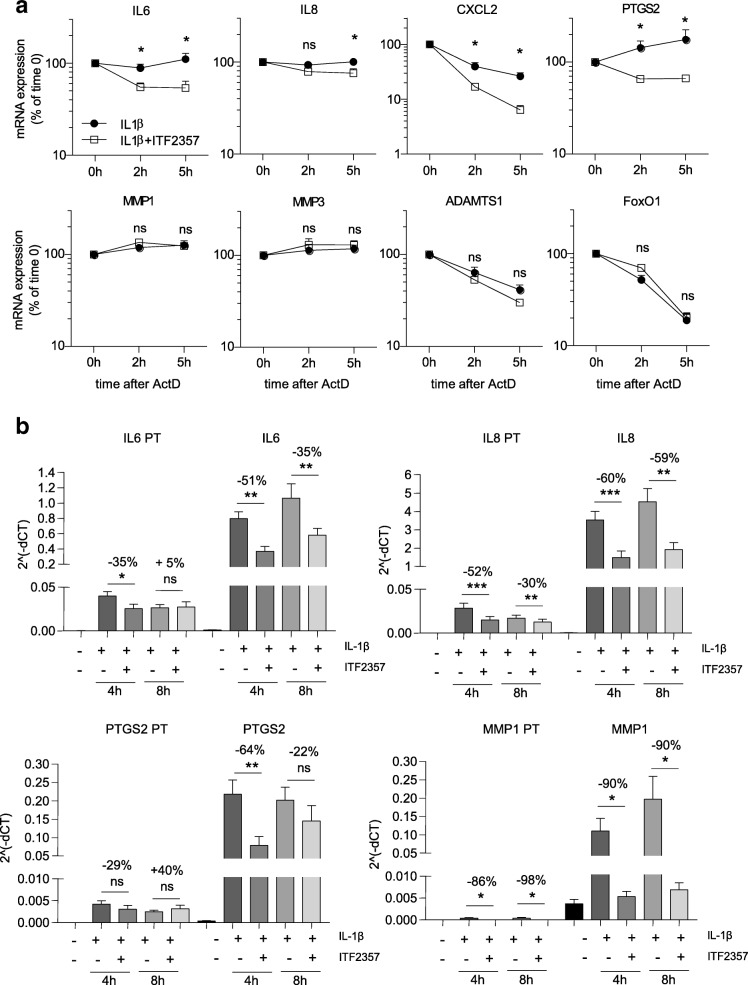
ITF2357 accelerates the mRNA decay of inflammatory markers. **a** Rheumatoid arthritis (RA) fibroblast-like synoviocytes (FLS) (*n* = 6) were left untreated or were treated with ITF2357 prior to incubation with IL-1β for 2 h. Transcription was blocked with actinomycin D (ActD) and RNA extracted at the indicated time points. Graphs show representative genes with enhanced mRNA degradation in the presence of ITF2357 (top panel), and examples of genes that were not regulated by ITF2357 at the level of transcript stability (bottom panel): *p < 0.05, Wilcoxon matched pairs test. **b** RA FLS (*n* = 7) were left untreated or were treated with ITF2357 prior to incubation with IL-1β for 4 and 8 h. The expression of primary transcripts (PT) and mature transcripts of *IL6*, *IL8, PTGS2* and *MMP1* were assessed by qPCR. Results are presented as 2^^(-ΔCT)^ of the targets of interest normalized to *GAPDH* housekeeping gene. Percentages indicate the average suppression caused by the presence of ITF2357 in the 7 independent experiments: *p < 0.05, **p < 0.01, ***p < 0.001, one-way analysis of variance with Greenhouse-Geisser correction followed by Fisher’s least significant difference test

In a previous study we demonstrated that both the pan-HDACi ITF2357 and trichostatin A (TSA) accelerate the mRNA decay of *IL6* in RA FLS and healthy donor macrophages [22]. To test whether this observation could be extended to other inflammatory mediators, we analyzed mRNA stability of the genes screened in our mRNA kinetics experiment. In addition to *IL6* mRNA, the stability of other transcripts, such as *IL8, CXCL2, PTGS2, ADAMTS1,* and *BCL2L1*, was reduced after ITF2357 treatment (Additional file 2). In contrast, other genes including *MMP1*, *MMP3*, *CXCL5, CXCL6, CXCL10*, *FoxO1* and *ADAMTS1* were not affected by ITF2357 at the post-transcriptional level, even though their mRNA expression was reproducibly regulated by ITF2357 (Additional files 1 and 2). Independent qPCR assays confirmed reduced mRNA stability of *IL6*, *IL8*, *CXCL2* and *PTGS2* transcripts after ITF2357 treatment (Fig. 2a).

In order to further dissect the transcriptional and post-transcriptional effects of ITF2357 on mRNA expression, we quantified unspliced primary transcripts of the genes that were either affected (*IL6*, *IL8* and *PTGS2*) or unaffected (*MMP1*) by ITF2357 at the level OF mRNA decay, and compared them with their respective mature transcripts (Fig. 2b). The difference between primary and mature transcript expression of *IL6*, *IL8* and *PTGS2* after 4 h treatment with ITF2357 was modest; however, it became more prominent at 8 h and marked a pronounced reduction in the expression of the mature transcript. Conversely, *MMP1* primary and mature transcript rates were similar. These results confirmed that genes found unaffected by ITF2357 in terms of mRNA decay (e.g. *MMP1*) are predominantly regulated at the transcriptional level, while those destabilized by ITF2357 treatment are post-transcriptionally regulated.

### ITF2357 leads to TTP transcriptional and post-translational changes

**Fig. 3:**
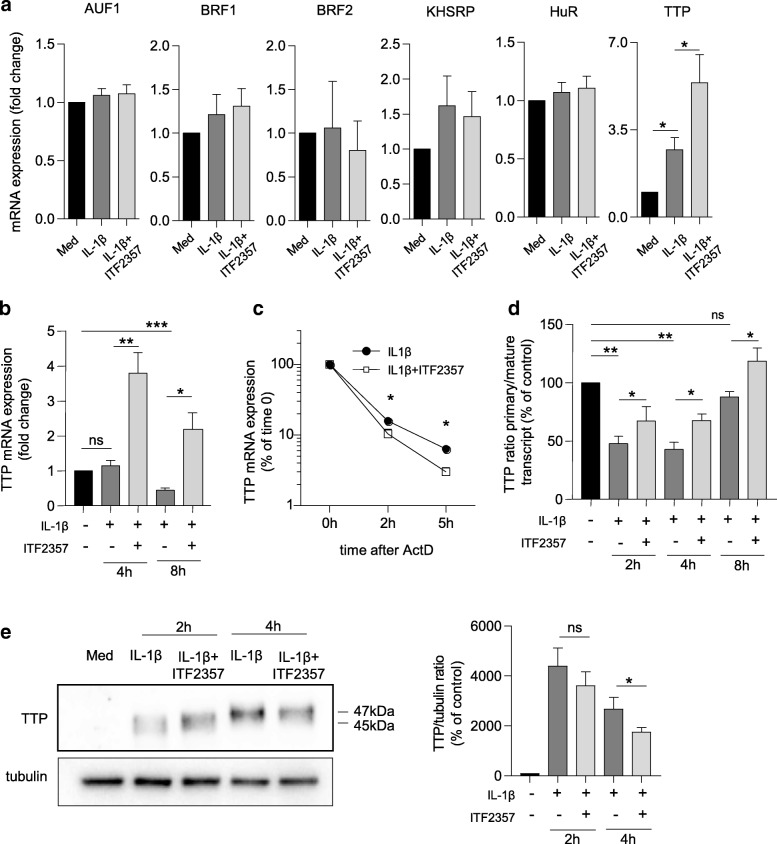
Tristetraprolin (TTP) expression and activity are induced by ITF2357. **a**, **b** Rheumatoid arthritis (RA) fibroblast-like synoviocytes (FLS) were left untreated or were treated with ITF2357 before incubation with IL-1β for either 2 h (**a**, *n* = 4) or for 4 and 8 h (**b**, *n* = 6). The mRNA expression of the Adenosine uridine-rich elements (ARE) binding proteins (ARE-BP) was analyzed by qPCR. **c** RA FLS (*n* = 6) were subject to mRNA stability analysis as specified in Fig. 2a and TTP expression was analyzed by qPCR: *p < 0.05, Wilcoxon matched pairs test. **d** RA FLS (*n* = 4) were treated with ITF2357 before incubation with IL-1β for either 2 h, 4 h or 8 h and the expression levels of TTP primary and mature transcript were analyzed by qPCR. Graph shows the 2^^(-ΔCT)^ ratio of the primary/mature transcript normalized to *GAPDH* housekeeping gene. **e** RA FLS were left untreated or were treated with ITF2357 before incubation with IL-1β for 2 and 4 h. Protein lysates were processed for immunoblotting with antibodies recognizing TTP and tubulin (left panel) and signal intensity (*n* = 8) was subsequently quantified by densitometry analysis (right panel). Band with higher apparent molecular mass corresponds to the post-translationally modified protein. **d**, **e** *p < 0.05, **p < 0.01, one-way analysis of variance with Greenhouse-Geisser correction followed by Fisher’s least significant difference test. ActD, actinomycin D; AUF, AU-rich binding factor; KHSRP, KH-type splicing regulatory protein; HuR, Hu antigen R

Early studies in cancer cells indicated that HDACi modulate ARE-BP function [28, 29]. Therefore, we investigated the expression of both destabilizing (TTP, AUF1, BRF1, BRF2, KHRSP) and stabilizing (HuR) ARE-BP after short treatment with ITF2357 in RA FLS. We found that TTP was induced by IL-1β and further upregulated by ITF2357. Conversely, other ARE-BP were either not induced by IL-1β or did not exhibit a specific regulation pattern in combination with the HDACi (Fig. 3a). We confirmed that ITF2357 led to sustained TTP mRNA expression at later time points (Fig. 3b), while not inducing any of the other ARE-BP (data not shown). We thus investigated whether induction of TTP mRNA expression by ITF2357 could also derive from the enhanced stability of the transcript. Surprisingly, ITF2357 rather acted as a destabilizing factor of TTP mRNA (Fig. 3c), while enhancing TTP primary transcript at all time points (Fig. 3d).

Following inflammatory or growth factor-driven p38 MAPK activation, TTP protein is phosphorylated on different amino acid residues, becoming inactivated [30]. We therefore examined whether ITF2357 could also affect TTP activity by altering its post-translational status. After 2 h IL-1β stimulation, we observed increased intensity of an immunoreactive band (45 kDa) at the expected mobility for TTP (Fig. 3e). A band with higher molecular weight of approximately 47 kDa was detected after 4 h, and confirmed by higher immunoblot resolution (Additional file 3A), suggesting phosphorylation of the protein [31]. ITF2357 significantly reduced the intensity of the higher band (Fig. 3e and Additional file 3A), similarly to p38 MAPK inhibition (Additional file 3B). Additionally, protein lysate treatment with λ-phosphatase reduced the intensity of TTP band upon IL-1β stimulation but did not further reduce TTP signal in samples treated with either ITF2357 or p38 inhibitor (Additional file 3C). Together, these results suggest that ITF2357 has a dual role in the regulation of TTP, by inducing mRNA expression at the transcriptional level, and by preventing TTP phosphorylation and subsequent inactivation.

### ITF2357 suppresses cytokine production independently of AUF1, BRF1, BRF2, KHSRP and HuR

**Fig. 4:**
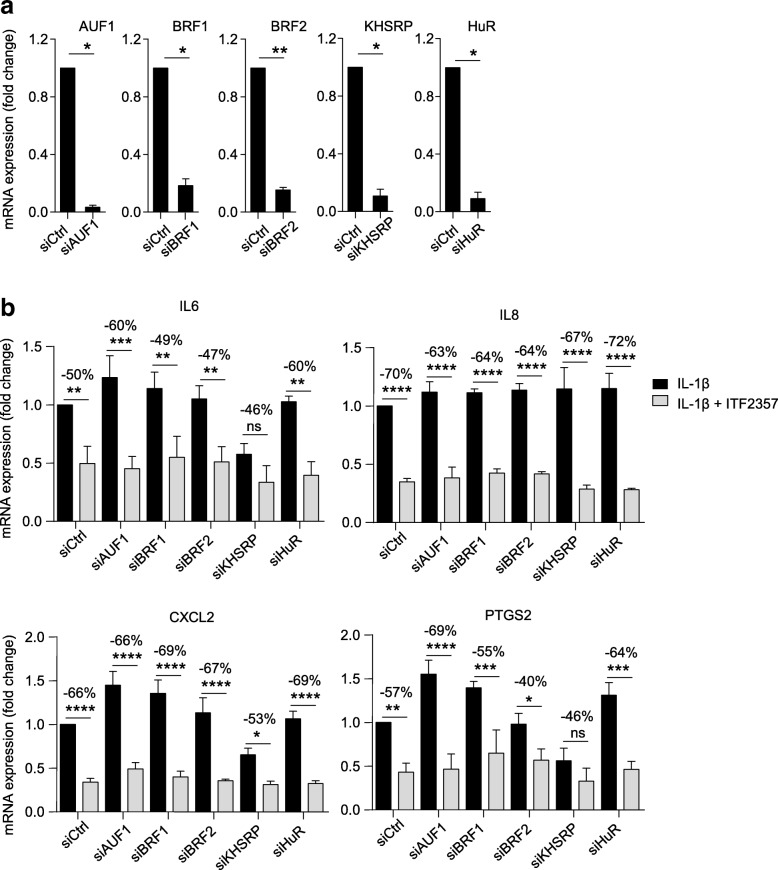
Knockdown of adenosine uridine-rich elements binding proteins does not cause changes in the expression of inflammatory mediators. **a** Rheumatoid arthritis (RA) fibroblast-like synoviocytes (FLS) (*n* = 3) were left untransfected or were transfected for 72 h with 20 nM control non-targeting siRNA (siCtrl) or 20 nM specific siRNA targeting *AUF1* (siAUF1), *BRF1* (siBRF1), *BRF2* (siBRF2), *KHSRP* (siKHSRP) and *HuR* (siHuR). Knockdown efficiency was verified at the mRNA level by qPCR. Data are presented as fold expression of mRNA compared to siCtrl transfected cells:*p < 0.05, **p < 0.01, ratio *t* test. **b** RA FLS (n = 3) were transfected as in **a** and further left untreated or treated with ITF2357, prior to stimulation with IL-1β for 8 *h. IL6*, *IL8*, *CXCL2* and *PTGS2* expression were determined by qPCR. Data are presented as fold expression of mRNA compared to siCtrl-treated IL-1β stimulated cells: *p < 0.05, **p < 0.01, ***p < 0.001, ****p < 0.0001, one-way analysis of variance followed by Fisher’s least significant difference test

Despite the prevalent effect of ITF2357 on TTP mRNA induction, we could not exclude the possibility that ITF2357 may impact other ARE-BP by different mechanisms of action, e.g. by modifying their post-translational state. To investigate whether any of the other ARE-BP included in our study would be required for ITF2357 effects on *IL6*, *IL8*, *CXCL2* or *PTGS2* mRNA expression, we performed knockdown of AUF1, BRF1, BRF2, KHSRP and HuR in RA FLS, achieving 80–95% silencing efficiency (Fig. 4a). We observed that knockdown of these genes led to minor changes in cytokine gene expression (Fig. 4b) and that ITF2357 similarly suppressed cytokine production in both control and ARE-BP knockdown conditions. A trend towards downregulation of *IL6* and *PTGS2* mRNA expression was noticeable upon KH-type splicing regulatory protein (KHSRP) knockdown, possibly indicating alternative mechanisms of regulation by this ARE-BP, which go beyond direct control of mRNA stability [32]. Overall, these results indicate that, despite potential post-translational modifications occurring on other ARE-BP after ITF2357 treatment, these would not be sufficient to mediate changes in the expression of the inflammatory mediators considered in our study.

### TTP silencing causes pro-inflammatory responses in RA FLS and is required to prevent ITF2357-dependent *IL6* suppression in murine fibroblasts

**Fig. 5:**
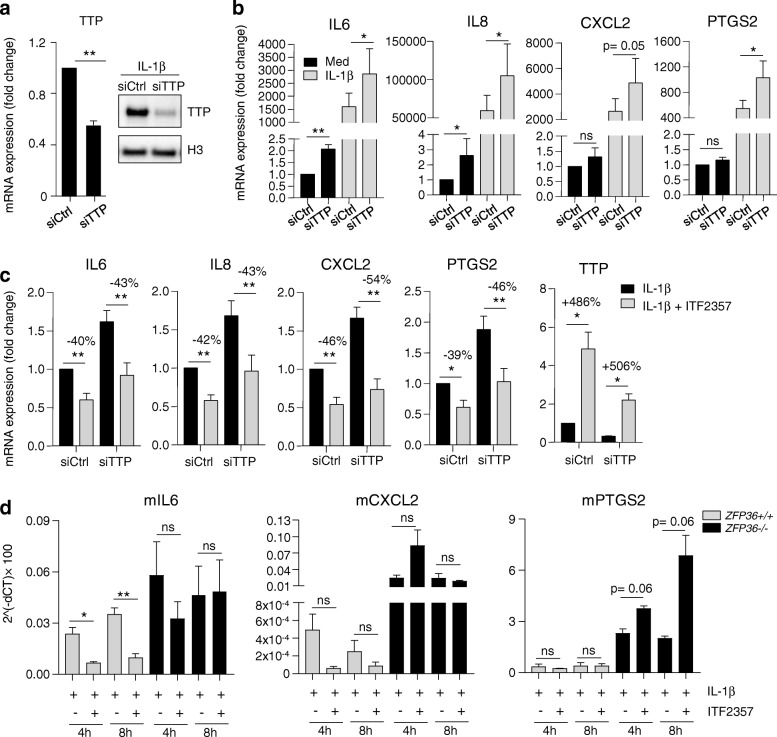
Tristetraprolin (TTP) silencing in rheumatoid arthritis (RA) fibroblast-like synoviocytes (FLS) and murine fibroblasts induces cytokine expression. **a** RA FLS (*n* = 4) were left untransfected or were transfected with control non-targeting siRNA (siCtrl) or specific siRNA targeting TTP (siTTP). Knockdown efficiency was verified at the mRNA level by qPCR (left panel) and by immunoblotting (right panel). For immunoblot samples, cells were stimulated with IL-1β for 2 h. Protein lysate was analyzed with antibodies recognizing TTP or control H3: **p < 0.01, ratio t-test. **b** RA FLS (*n* = 7) were transfected as in **a**, and further left untreated or treated with ITF2357, prior to stimulation with IL-1β for 8 h*.* Gene expression was determined by qPCR. Data are presented as fold change in mRNA expression compared to siCtrl conditions. **c** RA FLS were processed as in **b**, *IL6*, *IL8*, *CXCL2* and *PTGS2* (*n* = 7) and TTP expression (*n* = 3) was determined by qPCR. Data presented as fold change in mRNA expression compared to siCtrl-IL-1β stimulated conditions. **d** Wild-type (*ZFP36*^*+/+*^) or TTP knockout (*ZFP36*^*−/−*^) murine fibroblasts (*n* = 4) were either left untreated or treated with ITF2357 and were further stimulated with IL-1β. Gene expression was determined by qPCR. Results are presented as 2^^(-ΔCT)^× 100 of the target of interest normalized to *ACTB* housekeeping gene. **b-d** *p < 0.05, **p < 0.01, one-way analysis of variance with Greenhouse-Geisser correction followed by Fisher’s least significant difference test

We next investigated the effects of TTP silencing on cytokine expression in RA FLS. TTP siRNA-mediated knockdown resulted in 50% reduction of TTP mRNA expression (Fig. 5a, left panel), confirmed at the protein level (Fig. 5a, right panel), which was sufficient to cause increased cytokine expression of *IL6*, *IL8*, *CXCL2* and *PTGS2* (Fig. 5b), but not *MMP1* (data not shown) both in unstimulated (*IL6*, *IL8*) and IL-1β-stimulated conditions (*IL6*, *IL8, PTGS2*). Although not statistically significant (*p* = 0.05), induction of *CXCL2* after TTP knockdown was also observed. ITF2357 still mediated cytokine suppression in the presence or absence of TTP (Fig. 5c), possibly because TTP silencing only reduced the steady-state levels of TTP mRNA but was unable to fully prevent its upregulation by ITF2357 (Fig. 5c, right panel). In line with this hypothesis, recent studies indicated that, even when minimal, TTP expression is sufficient to suppress inflammatory responses [30].

Because TTP protein domains are remarkably conserved within vertebrates, and share a common regulatory mechanism [33, 34], we made use of fibroblasts derived from wild-type (*ZFP36*^+/+^) and TTP knockout (*ZFP36*^−/−^) mice [35], and stimulated them with IL-1β to mimic experimental conditions used in RA FLS. Higher expression of *IL6*, *CXCL2* and *PTGS2* mRNA in *ZFP36*^−/−^ fibroblasts indicated that these cytokines are TTP targets, as previously described [36–38]. In addition, while in wild-type fibroblasts *IL6* mRNA expression was significantly reduced by ITF2357, no significant suppression was observed in *ZFP36*^−/−^ fibroblasts (Fig. 5d). A similar trend was also observed for *CXCL2*, while *PTGS2* was not reduced by ITF2357 in wild-type fibroblasts. Our findings indicate that TTP is an important regulator of cytokine mRNA expression in RA FLS, and suggest that this ARE-BP is responsible for mediating part of the anti-inflammatory properties of ITF2357.

## Discussion

In RA and in other immune-mediated inflammatory diseases (IMIDs), the excessive production and accumulation of cytokines and chemokines contributes to the perpetuation of chronic inflammation and immune responses [1]. Continuous exposure to pro-inflammatory stimuli drives RA FLS to develop an epigenetically imprinted, aggressive phenotype and inflammatory memory that promotes the degradation of synovial joints [39]. Development of immunomodulatory epigenetic inhibitors has emerged in recent years. Among these, HDACi comprise a class of small anti-inflammatory molecules that showed pre-clinical potential for the treatment of RA [18]. Despite extensive research in recent years, the mode of action of these compounds remains largely unknown.

Here we show that in RA FLS, pan-HDACi ITF2357 efficiently suppresses cytokine production independently of the kinetics of gene induction by IL-1β. As we previously found that IL-6, a key cytokine contributing to RA pathobiology, was suppressed by ITF2357 via acceleration of *IL6* mRNA decay [22], we aimed to investigate whether post-transcriptional, rather than transcriptional, regulatory events could be the key factor explaining the broad anti-inflammatory effects of ITF2357. Of note, recent reports indicate that prolonged exposure to TNF leads to a gradual reshaping of the FLS transcriptome, which is largely dependent on mRNA stability processes [14], highlighting the importance of post-transcriptional regulatory mechanisms in maintaining the chronicity of inflammation in RA. We extended our analysis to additional IL-1β-induced cytokines (*IL8, CXCL2*) and mediators of inflammatory responses, matrix degradation, and cell survival (*PTGS2, ADAMTS1,* and *BCL2L1*). We confirmed that a subset of these genes, specifically *IL8*, *CXCL2* and *PTGS2*, were subject to mRNA stability regulation by ITF2357. On the contrary, some targets displayed sustained stability (*MMP1–3, CXCL10, CXCL5–6)*, while others rapidly decayed over time but were not further destabilized by ITF2357 (*IRF1, FoxO1*). More intriguingly, kinetics played an important role in the transcriptional or post-transcriptional regulation of genes affected by ITF2357. Indeed, while mostly affected at the transcriptional level after shorter exposure to ITF2357, the mRNA expression of *IL6*, *IL8* and *PTGS2* was post-transcriptionally regulated at later time points. These results indicate that the initial anti-inflammatory events mediated by ITF2357 occur by suppressing the nascent production of cytokine mRNA, while subsequent immune suppressive functions are related to the destabilization of their transcripts.

A key mechanism responsible for the post-transcriptional regulation of gene expression is ARE-BP-mediated mRNA decay [4]. We evaluated whether ARE-BP could mediate the effects of ITF2357 on cytokine mRNA stability in RA FLS and found that TTP mRNA expression rapidly increased after short exposure to IL-1β. After treatment with ITF2357, TTP mRNA was not stabilized despite being induced at all time-points, implying that a transcriptional component is responsible for the regulation of this ARE-BP by HDACi. To date, the mRNA-destabilizing TTP has been best described as a regulator of inflammatory processes [40]. TTP expression is significantly increased in the synovial joints in RA compared to non-inflamed joints, and it is abundant in macrophages and synovial fibroblasts [41], possibly indicating a relevant role for this ARE-BP in these cells. Studies of peripheral blood mononuclear cells in RA have reported a global reduction of TTP expression, compared to healthy controls and patients with osteoarthritis (OA) [12, 42]. In animal studies, knockout of TTP has been shown as sufficient to the development of a complex inflammatory phenotype, characterized by auto-immunity and polyarthritis [10] On the contrary, induction of TTP expression is protective in collagen-induced arthritis (CIA) [13].

In our study, we observed that besides affecting the transcriptional regulation of TTP, ITF2357 additionally reduced the abundance of a higher molecular-weight form of TTP. Treatment with phosphatase confirmed this as the phosphorylated form of the protein. The destabilizing effects of ITF2357 on TTP mRNA decay further support this finding, as dephosphorylated and active TTP would also cause its own mRNA to be degraded [13]. The equilibrium between the phosphorylated and the dephosphorylated pools of TTP has frequently been reported to be a critical feature in the determination of the inflammatory response [30]. Mice expressing a phosphorylation-deficient form of TTP, in which serines 52 and178 are converted to arginine residues, are protected from CIA, as a consequence of increased functionality of the protein [11, 30]. Also, activation or depletion of phosphatases that revert TTP to its dephosphorylated form, such as PP2A and Dusp1, reduce the production of cytokines such as *IL6, IL8* and *TNF*, and increase broad pro-inflammatory gene expression, respectively [11, 43, 44].

Phosphorylation is the most common post-translational modification of TTP and other ARE-BP, but other modifications have been reported [45, 46]. Thus, it remains possible that ITF2357 may enhance the acetylation levels of TTP and subsequently reduce its phosphorylation. Indirect regulation of TTP phosphorylation by HDACi is yet another possibility. In fact, evidence from the literature suggests that HDAC1,2 and 3 can bind to and acetylate Dusp1 [47]. On the contrary, direct effects of HDACi on p38 activation are likely to be excluded, as we found pan-HDACi to leave p38 phosphorylation unaltered in IL-1β-stimulated FLS [22]. Similarly, ITF2357 may affect mRNA stability independently of the c-Jun N-terminal kinase (JNK) signaling pathway, as *FoxO1* mRNA stability, previously shown to be mediated by JNK inhibition [48] was not affected by ITF2357.

We tested whether single silencing of multiple ARE-BP could result in the differential regulation of *IL6*, *IL8, CXCL2* and *PTGS2*. Unlike other ARE-BP, TTP silencing caused increased expression of inflammatory mediators and proved to be a critical regulatory factor in RA FLS. Additionally, ITF2357 reduced *IL6* and *CXCL2* production in *ZFP36*^+/+^ but not *ZFP36*^−/−^ murine fibroblasts, overall demonstrating crucial involvement of TTP in *IL6* regulation by HDACi.

## Conclusions

Recent studies suggested that the induced expression and the reduced phosphorylation of TTP could be beneficial in dampening inflammatory responses in arthritis [11]. Our results indicate that ITF2357 functions as an activator of TTP function and transcription in RA FLS, and provide novel understanding of how HDACi dictate not only the transcriptional, but also the post-transcriptional regulation of inflammatory genes. These findings provide rationale for further evaluation of HDACi as a therapeutic tool in RA and other chronic inflammatory diseases.

## Additional files

Additional file 1: **Figure S1.** Kinetics of mRNA regulation by ITF2357. (PDF 293 kb)

Additional file 2: **Figure S2.** Effects of ITF2357 on mRNA stability. (PDF 816 kb)

Additional file 3: **Figure S3.** TTP post-translational changes after ITF2357 treatment. (PDF 283 kb)

Additional file 4: Sequences of primers. (PDF 111 kb)

## Abbreviations

ActD: Actinomycin D
ARE: Adenosine uridine-rich elements
ARE-BP: ARE-binding proteins
AUF1: AU-rich binding factor-1
DMEM: Dulbecco’s Modified Eagle Medium
FBS: Fetal bovine serum
FLS: Fibroblast-like synoviocytes
HDACi: Histone deacetylase inhibitor
HuR: Hu antigen R
IL: Interleukin
JNK: c-Jun N-terminal kinase
kDa: KilotDalton
KHSRP: KH-type splicing regulatory protein
MAPK: Mitogen-activated protein kinase
MMP: Matrix metalloproteinase
mRNA: messenger RNA
PT: Primary transcripts
RA: Rheumatoid arthritis
TNF: Tumor necrosis factor
TSA: Trichostatin A
TTP: Tristetraprolin
