# Supplementary material for: Control of cytokine mRNA degradation by the histone deacetylase inhibitor ITF2357 in rheumatoid arthritis fibroblast-like synoviocytes: beyond transcriptional regulation
Source: Arthritis Res Ther. 2018 Jul 20;20:148. doi: 10.1186/s13075-018-1638-4 (PMC6053802; doi:10.1186/s13075-018-1638-4)
Supplement: Supplementary file 1 — Figure S1. Kinetics of mRNA regulation by ITF2357. (PDF 293 kb) [file 13075_2018_1638_MOESM1_ESM.pdf]

Figure S1

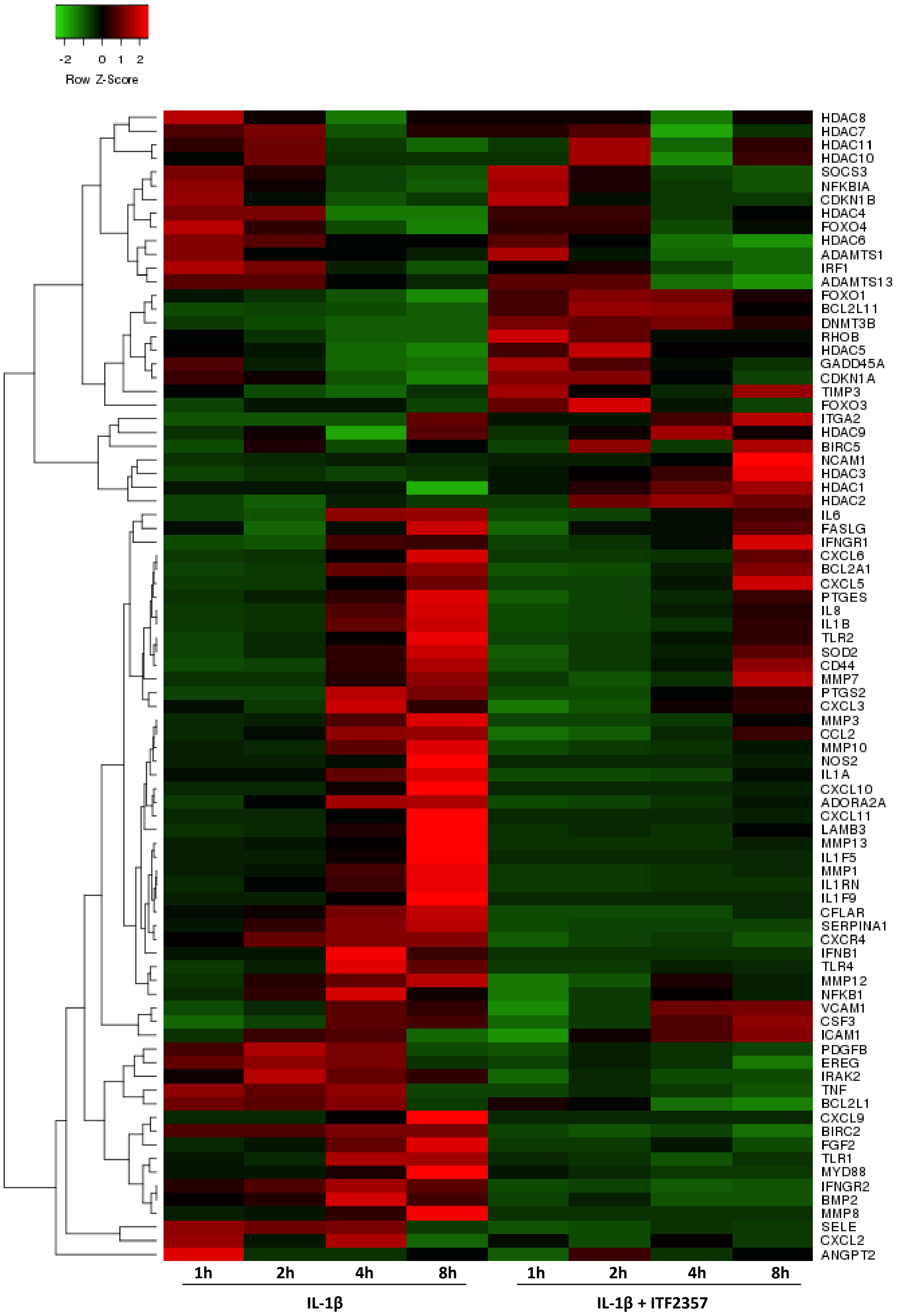

Figure S1. Kinetics of mRNA regulation by ITF2357. RA FLS were either left untreated or were treated with ITF2357 prior to incubation with IL-1β for the indicated time. Temporal changes in mRNA accumulation of IL-1β-inducible genes were monitored using a customized qPCR array. Data are presented as Z-scores of mean (n=3) fold changes relative to unstimulated cells and hierarchically clustered with the average linkage method.
