# Supplementary material for: Control of cytokine mRNA degradation by the histone deacetylase inhibitor ITF2357 in rheumatoid arthritis fibroblast-like synoviocytes: beyond transcriptional regulation
Source: Arthritis Res Ther. 2018 Jul 20;20:148. doi: 10.1186/s13075-018-1638-4 (PMC6053802; doi:10.1186/s13075-018-1638-4)
Supplement: Supplementary file 2 — Figure S2. Effects of ITF2357 on mRNA stability. (PDF 816 kb) [file 13075_2018_1638_MOESM2_ESM.pdf]

Figure S2

A

Gene target	IL-1β ActD 2h	SEM	IL-1β +ITF2357 ActD 2h	SEM	IL-1β ActD 5h	SEM	IL-1β +ITF2357 ActD 5h	SEM
ADAMTS1	48%	6%	32%	4%	30%	4%	18%	3%
ADAMTS13	86%	10%	76%	6%	76%	6%	76%	8%
ADORA2A	98%	5%	91%	6%	88%	6%	79%	7%
ANGPT2	135%	5%	130%	24%	124%	20%	99%	17%
BCL2A1	103%	5%	101%	10%	94%	4%	88%	9%
BCL2L1	84%	4%	67%	4%	44%	7%	29%	5%
BCL2L11	29%	6%	19%	2%	15%	2%	12%	1%
BIRC2	81%	2%	67%	5%	53%	4%	39%	1%
BIRC5	89%	13%	84%	3%	87%	13%	79%	5%
BMP2	68%	2%	59%	4%	34%	6%	22%	3%
CCL2	78%	9%	70%	12%	75%	8%	59%	14%
CD44	104%	2%	99%	3%	100%	3%	99%	4%
CDKN1A	69%	5%	68%	5%	53%	6%	45%	5%
CDKN1B	15%	4%	8%	3%	5%	1%	3%	0%
CFLAR	101%	4%	88%	5%	82%	11%	69%	6%
CSF3	67%	10%	61%	21%	68%	15%	55%	20%
CXCL10	95%	10%	94%	6%	87%	5%	86%	6%
CXCL11	110%	11%	116%	41%	89%	12%	98%	38%
CXCL2	28%	12%	9%	4%	21%	12%	5%	3%
CXCL3	81%	6%	64%	9%	81%	9%	67%	16%
CXCL5	101%	12%	105%	8%	101%	18%	108%	11%
CXCL6	107%	4%	99%	2%	102%	6%	108%	6%
CXCL9	278%	207%	106%	48%	302%	256%	110%	54%
CXCR4	59%	9%	69%	10%	26%	8%	25%	5%
DNMT3B	66%	8%	40%	3%	51%	8%	33%	6%
EREG	95%	7%	86%	5%	81%	6%	81%	9%
FASLG	84%	10%	95%	5%	92%	12%	98%	5%
FGF2	89%	2%	85%	6%	75%	5%	71%	7%
FOXO1	36%	9%	38%	7%	9%	2%	6%	1%
FOXO3	75%	3%	72%	4%	40%	3%	34%	3%
FOXO4	82%	6%	92%	4%	74%	5%	76%	6%
GADD45A	36%	3%	25%	5%	19%	2%	9%	2%
HDAC1	96%	3%	100%	3%	97%	6%	102%	5%
HDAC10	90%	8%	77%	10%	78%	8%	74%	10%
HDAC11	94%	10%	98%	5%	85%	9%	92%	5%
HDAC2	82%	12%	68%	6%	71%	13%	65%	7%
HDAC3	94%	5%	89%	5%	91%	6%	86%	3%
HDAC4	81%	6%	83%	2%	63%	6%	61%	3%
HDAC5	81%	7%	66%	3%	73%	7%	58%	6%
HDAC6	88%	3%	88%	6%	79%	5%	73%	7%
HDAC7	73%	8%	63%	5%	51%	7%	41%	7%
HDAC8	92%	2%	90%	3%	85%	5%	73%	3%
HDAC9	101%	7%	89%	4%	70%	10%	74%	7%
ICAM1	90%	2%	91%	5%	82%	5%	76%	5%
IFNB1	97%	15%	84%	18%	66%	11%	56%	19%
IFNGR1	91%	12%	81%	11%	69%	5%	64%	7%
IFNGR2	111%	10%	93%	12%	99%	11%	99%	9%
IL1A	78%	13%	64%	7%	56%	15%	44%	13%
IL1B	81%	12%	73%	15%	80%	19%	77%	26%
IL1F5	91%	6%	73%	12%	74%	1%	77%	8%
IL1F9	69%	11%	132%	76%	43%	15%	77%	19%
IL1RN	88%	5%	122%	26%	95%	14%	72%	18%
IL6	56%	15%	31%	14%	51%	17%	26%	14%
IL8	70%	9%	53%	15%	71%	8%	49%	16%
IRAK2	57%	10%	46%	10%	27%	9%	16%	5%
IRF1	17%	4%	15%	4%	4%	1%	3%	0%
ITGA2	73%	6%	69%	13%	61%	6%	83%	12%
LAMB3	94%	2%	92%	6%	83%	7%	89%	4%
MMP1	96%	5%	103%	9%	88%	7%	105%	9%
MMP10	116%	15%	107%	7%	108%	17%	102%	5%
MMP13	141%	49%	107%	45%	99%	40%	105%	28%
MMP3	100%	3%	95%	2%	97%	2%	104%	3%
MMP7	84%	10%	114%	7%	83%	4%	103%	6%
MMP8	254%	200%	106%	40%	288%	217%	115%	74%
PTGES	102%	3%	101%	2%	107%	6%	102%	5%

MYD88	94%	5%	88%	4%	78%	7%	70%	4%
NCAM1	87%	7%	130%	35%	73%	9%	116%	41%
NFKB1	98%	2%	93%	4%	72%	4%	58%	7%
NFKBIA	2%	0%	1%	0%	1%	0%	0%	0%
NOS2	85%	8%	82%	13%	72%	6%	57%	17%
PDGFB	74%	10%	47%	12%	23%	8%	12%	4%
PTGS2	75%	13%	41%	13%	68%	16%	44%	19%
RHOB	23%	3%	20%	2%	3%	1%	1%	0%
SELE	65%	11%	44%	10%	46%	9%	36%	8%
SERPINA1	100%	5%	102%	4%	102%	10%	105%	7%
SOCS3	7%	1%	4%	0%	2%	0%	2%	0%
SOD2	100%	4%	97%	4%	98%	2%	93%	2%
TIMP3	131%	83%	21%	10%	73%	20%	36%	18%
TLR1	73%	5%	80%	6%	53%	6%	61%	9%
TLR2	111%	10%	102%	15%	98%	11%	89%	13%
TLR4	86%	5%	76%	3%	71%	5%	52%	3%
TNF	23%	13%	8%	3%	12%	9%	3%	1%
VCAM1	101%	3%	99%	5%	95%	6%	94%	8%
B2M	106%	5%	100%	1%	109%	3%	104%	3%
HPRT1	102%	2%	106%	4%	97%	4%	106%	2%
RPL13A	96%	1%	94%	2%	92%	2%	92%	2%
GAPDH	97%	3%	100%	7%	103%	6%	99%	3%
ACTB	100%	3%	101%	3%	101%	2%	100%	4%

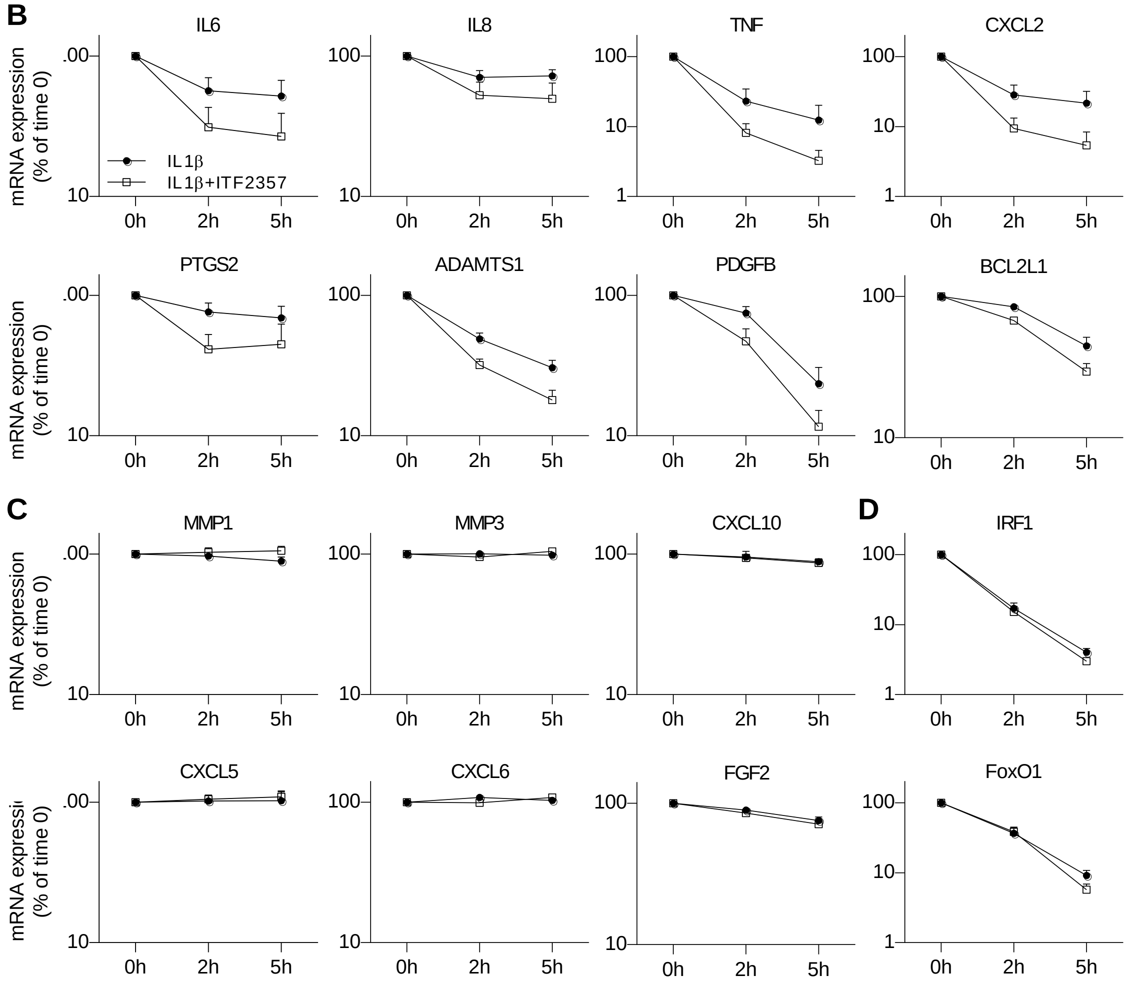

Figure S2. Effects of ITF2357 on mRNA stability. (A-B) RA FLS (n=4) were left untreated or were treated with ITF2357 prior to incubation with IL-1β for 2h. Transcription was then blocked with 10μg/ml of actinomycin D (ActD) and RNA extracted at the indicated time points from the start of ActD treatment. mRNA degradation in the presence or absence of ITF2357 was monitored using customized qPCR arrays. mRNA expression values for 0h time point were normalized to 100%. (A) Mean percentage ± SEM of remaining mRNA after 2h and 5h ActD treatment, calculated relative to 100% value of relative controls from 0h ActD treatment. (B-C-D) RA FLS (n=4) were treated as in A. Representative genes displaying enhanced mRNA degradationby ITF2357 (B), no appreciable decay regardless of IL-1β stimulation (C), or no destabilization by ITF2357 (D).
