# Supplementary material for: Control of cytokine mRNA degradation by the histone deacetylase inhibitor ITF2357 in rheumatoid arthritis fibroblast-like synoviocytes: beyond transcriptional regulation
Source: Arthritis Res Ther. 2018 Jul 20;20:148. doi: 10.1186/s13075-018-1638-4 (PMC6053802; doi:10.1186/s13075-018-1638-4)
Supplement: Supplementary file 3 — Figure S3. TTP post-translational changes after ITF2357 treatment. (PDF 283 kb) [file 13075_2018_1638_MOESM3_ESM.pdf]

Figure S3

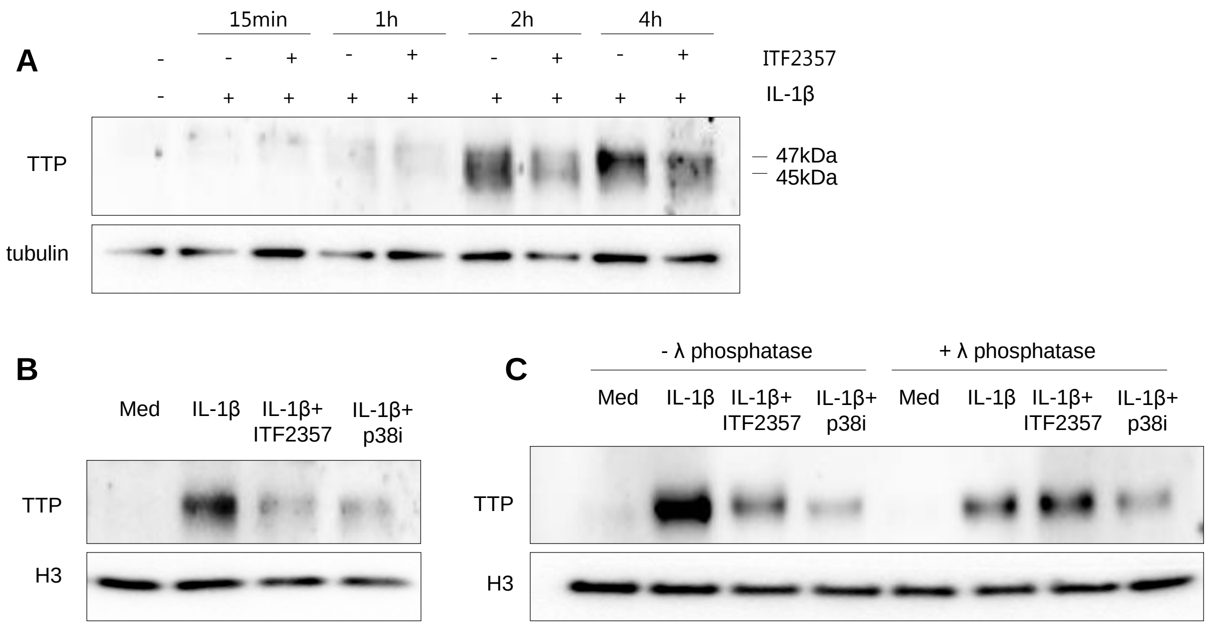

Figure S3. TTP post-translational changes after ITF2357 treatment. (A) FLS (n=2) were left untreated or were treated with ITF2357 prior to incubation with IL-1β for 15min, 1h, 2h and 4h. Protein lysates were immunoblotted with antibodies recognizing TTP and tubulin. Proteins detected at 45 and 47kDa correspond to TTP protein, and to its post-translationally modified counterpart, respectively. (B) FLS (n=2) were left untreated or were treated with either ITF2357 or p38 inhibitor (p38i, SB202190) prior to incubation with IL-1β for 4h, and immunoblotted for TTP and H3.(C) FLS (n=1) were treated as in (B) and protein lysate incubated either with or without λ phosphatase, displaying activity towards phosphorylated serine, threonine and tyrosine residues. Samples were immunoblotted as in (B).
