# Supplementary material for: Control of cytokine mRNA degradation by the histone deacetylase inhibitor ITF2357 in rheumatoid arthritis fibroblast-like synoviocytes: beyond transcriptional regulation
Source: Arthritis Res Ther. 2018 Jul 20;20:148. doi: 10.1186/s13075-018-1638-4 (PMC6053802; doi:10.1186/s13075-018-1638-4)
Supplement: Supplementary file 4 — Sequences of primers. (PDF 111 kb) [file 13075_2018_1638_MOESM4_ESM.pdf]

Table S1. Sequences of human primers used for qPCR analysis.

Gene	Forward primer	Reverse primer
GAPDH	GCCAGCCGAGCCACATC	TGACCAGGCGCCCAATAC
IL6	GACAGCCACTCACCTCTTCA	CCTCTTTGCTGCTTTCACAC
PT IL6	ACATCCTCGACGGCATCTCAG	CCCAGCAAAGACCTCCTAATG
IL8	GCTCTGTGTGAAGGTGCAGT	CCAGACAGAGCTCTCTTCCA
PT IL8	ATTGAGAGTGGACCACACTG	ACTACTGTAATCCTAACACCTG
CXCL2	GAAAGCTTGTCTCAACCCCG	AGTTGGATTTGCCATTTTTCAGC
PTGS2	ACTGCTCAACACCGGAATTT	TGCATTTCGAAGGAAGGGAA
PT PTGS2	GGTGCCACTTTCCACATTTT	AGGCTTCCCAGCTTTTGTAG
MMP1	CGGGGCTTTGATGTACCCTA	CGATGGGCTGGACAGGATTT
PT MMP1	GGCCACAAAGTTGATGCAGT	TTGCTACGGCAATGAAATGGAG
MMP3	GAGGACACCAGCATGAACCT	CACCTCCAGAGTGTCGGAGT
ADAMTS1	GTCTCACCAAAGGACAGGTG	GTACCATCTACAACCTTGGGC
FOXO1	TCTTCCTCCTGAGTCTGGGTAATT	CAGAGAGCTACCAAGGATTCATGA
AUF1	TTTGTTGGTGGCCTTTCTCC	ATTCCACCTCACCAAAACCAC
BRF1	ATGCAAGGGTAACAAGATGCTC	CACTGCCTTTCTGTCCAGC
BRF2	TCCAGAAACATGTCGACCAC	AGGGATTTCTCTGTCTTGCAC
KSRP	CTTACAAAGTGCAGCAAGCC	AGATCCGTACTCATTCCGGT
HuR	AAGCCTGTTCAGCAGCATTG	CCAAGCTGTGTCCTGCTACT
TTP	CTGCCATCTACGAGAGCCT	ACTCAGTCCCTCCATGGTC
PT TTP	GCCATCTACGAGGTGAGTCC	AGTTTGCGGCGCTAGAGAG

Table S2. Sequences of murine primers used for qPCR analysis.

Gene	Forward primer	Reverse primer
IL6	CAGTCAGGCAACAAAGATGGG	AACTTTCCCTCACCCTAGCAG
CXCL2	TCCAGAGCTTGAGTGTGACG	GCAAACTTTTTGAACGCCCT
PTGS2	ACTTACAGGAGAGAAGGAAATGGC	AGGTTTTTCCACCAGCAGGG
ACTB	TTCTTTGCAGCTCCTTCGTT	CGATGGAGGGGAATACAGC
